# Polygenic Markers in Patients Diagnosed of Autosomal Dominant Hypercholesterolemia in Catalonia: Distribution of Weighted LDL-c-Raising SNP Scores and Refinement of Variant Selection

**DOI:** 10.3390/biomedicines8090353

**Published:** 2020-09-15

**Authors:** Jesús M. Martín-Campos, Sheila Ruiz-Nogales, Daiana Ibarretxe, Emilio Ortega, Elisabet Sánchez-Pujol, Meritxell Royuela-Juncadella, Àlex Vila, Carolina Guerrero, Alberto Zamora, Cristina Soler i Ferrer, Juan Antonio Arroyo, Gemma Carreras, Susana Martínez-Figueroa, Rosa Roig, Núria Plana, Francisco Blanco-Vaca, Xarxa d’Unitats de Lípids i Arteriosclerosi (XULA)

**Affiliations:** 1 Metabolic Bases of Cardiovascular Risk Group, Santa Creu i Sant Pau Hospital Research Institute (IR-HSCSP)—Sant Pau Biomedical Research Institute (IIB-Sant Pau), 08041 Barcelona, Spain; genetica.ruiz@fundacionimo.org; 2 Spanish Biomedical Research Center in Diabetes and Associated Metabolic Disorders (CIBERDEM), 28029 Madrid, Spain; daianaig@hotmail.com (D.I.); RRoig@santpau.cat (R.R.); nplana@grupsagessa.com (N.P.); 3 Research Unit on Lipids and Atherosclerosis, Vascular Medicine and Metabolism Unit, Sant Joan University Hospital, Rovira i Virgili University, Pere Virgili Health Research Institute (IISPV), 43204 Reus, Spain; 4 Endocrinology and Nutrition Service, Hospital Clínic, 08036 Barcelona, Spain; EORTEGA1@clinic.cat; 5 Spanish Biomedical Research Center in Physiopathology of Obesity and Nutrition (CIBEROBN), 28029 Madrid, Spain; 6 Internal Medicine Service, Hospital-Asil de Granollers, Granollers, 08402 Barcelona, Spain; esanchezpujol@gmail.com; 7 Internal Medicine Service, Altahia, Xarxa Assistencial Universitària de Manresa, Manresa, 08243 Barcelona, Spain; mroyuelajuncadella@gmail.com; 8 Internal Medicine Service, Hospital de Figueres, Figueres, 17600 Girona, Spain; avila@salutemporda.cat; 9 Internal Medicine Service, Hospital de Terrassa-Consorci Sanitari de Terrassa, Terrassa, 08227 Barcelona, Spain; carolina.guerrero.b@gmail.com; 10 Internal Medicine Service, Hospital Sant Joan de Déu de Martorell, Martorell, 08760 Barcelona, Spain; 11 Internal Medicine Service, Corporació de Salut del Maresme i La Selva, Hospital de Blanes, Blanes, 17300 Girona, Spain; azamora@salutms.cat; 12 Internal Medicine Service, Hospital Santa Caterina, Salt, 17190 Girona, Spain; cristina.soler@ias.cat; 13 Internal Medicine Service, Santa Creu i Sant Pau Hospital (HSCSP)-IIB Sant Pau, 08041 Barcelona, Spain; JArroyo@santpau.cat; 14 Pediatric Service, HSCSP-IIB Sant Pau, 08041 Barcelona, Spain; GCarreras@santpau.cat; 15 Department of Pediatrics, Obstetrics and Gynecology and of Preventive Medicine and Public Health, Autonomous University of Barcelona (UAB), Cerdanyola del Valles, 08193 Barcelona, Spain; 16 Biochemistry Service, HSCSP-IIB Sant Pau, 08041 Barcelona, Spain; SMartinezF@santpau.cat; 17 Biochemistry and Molecular Biology Department, UAB, Cerdanyola del Valles, 08193 Barcelona, Spain

**Keywords:** familial hypercholesterolemia, atherosclerosis, genetic risk scores, cardiovascular risk, molecular diagnosis

## Abstract

Familial hypercholesterolemia (FH) is associated with mutations in the low-density lipoprotein (LDL) receptor (*LDLR*), apolipoprotein B (*APOB*), and proprotein convertase subtilisin/kexin 9 (*PCSK9*) genes. A pathological variant has not been identified in 30–70% of clinically diagnosed FH patients, and a burden of LDL cholesterol (LDL-c)-raising alleles has been hypothesized as a potential cause of hypercholesterolemia in these patients. Our aim was to study the distribution of weighted LDL-c-raising single-nucleotide polymorphism (SNP) scores (weighted gene scores or wGS) in a population recruited in a clinical setting in Catalonia. The study included 670 consecutive patients with a clinical diagnosis of FH and a prior genetic study involving 250 mutation-positive (FH/M+) and 420 mutation-negative (FH/M−) patients. Three wGSs based on LDL-c-raising variants were calculated to evaluate their distribution among FH patients and compared with 503 European samples from the 1000 Genomes Project. The FH/M− patients had significantly higher wGSs than the FH/M+ and control populations, with sensitivities ranging from 42% to 47%. A wGS based only on the SNPs significantly associated with FH (wGS8) showed a higher area under the receiver operating characteristic curve, and higher diagnostic specificity and sensitivity, with 46.4% of the subjects in the top quartile. wGS8 would allow for the assignment of a genetic cause to 66.4% of the patients if those with polygenic FH are added to the 37.3% of patients with monogenic FH. Our data indicate that a score based on 8 SNPs and the75th percentile cutoff point may identify patients with polygenic FH in Catalonia, although with limited diagnostic sensitivity and specificity.

## 1. Introduction

Familial hypercholesterolemia (FH) is an autosomal codominant disease characterized by elevated plasma low-density lipoprotein (LDL) cholesterol (LDL-c) >4.9 mmol/L and high risk of premature cardiovascular disease (CVD) [1]. It is mainly due to the loss of function variants in the LDL receptor gene (*LDLR*) (OMIM #143890), and includes variants in other genes encoding proteins that interact with LDLR, such as the LDLR ligand apolipoprotein B-100 (*APOB*) (OMIM #144010) and the LDLR catabolic regulator proprotein convertase subtilisin/kexin type 9 (*PCSK9*) (OMIM #603776). Considering the FH phenotype, the overall prevalence of FH in Catalonia was estimated as 0.52% [2], corresponding to one in 192 individuals. This estimate is higher than the worldwide FH prevalence recently calculated to be 0.32% (corresponding to one in 313 individuals) [3,4]. It is noteworthy that the estimated prevalence shows wide variations depending on the diagnostic criteria applied, both among countries and within them [3], and that less than 10% of countries have reported FH prevalence in their general populations [4].

In Europe, the clinical diagnosis of FH is mainly scored by applying the Dutch Lipid Clinic Network (DLCN) [5] criteria, the most widely used method worldwide [6], while other countries use the Simon Broome (SB) [7] criteria. Both include personal and family histories of CVD, hypercholesterolemia, and the presence of tendinous xanthomata and/or arcus cornealis. However, improvements in hypercholesterolemia therapy, such as the wider use of statins in FH families and CVD patients over the past 30 years and the implementation of public awareness campaigns about the dangers of an unhealthy lifestyle have decreased the average LDL-c in the general populations of some countries [8], making part of the clinical criteria less useful [9,10]. Thus, genetic diagnosis is increasingly considered as the “gold standard” for FH diagnosis [1,3] in order to differentiate it from phenocopies due to other pathologies.

Plasma lipid levels, as quantitative traits, are influenced by common multiple susceptibility variants in several genes involved in lipoprotein metabolism [11]. Such genes are mainly identified in genome-wide association studies (GWASs) [12]. For example, an LDL-c risk score using 10 single-nucleotide polymorphisms (SNPs) showing genome-wide significance explained 3.4% of the total variance in LDL-c in three European cohorts [13], and a weighted score combining six common genetic variants, each with a small effect, were highly predictive of circulating lipid levels in the Framingham Heart Study [14]. Also, genetic scores showed association with subclinical and clinical cardiovascular outcomes [15].

Recently, some studies using different weighted LDL-c gene scores (wGSs) showed that 20–80% of mutation-negative FH patients presented a high score [16,17,18,19,20,21], reflecting a burden of common LDL-c-raising genetic variants with respect to the control population. Although each variant has a modest effect on LDL-c, the accumulation of these variants can lead to a phenotype similar to that of monogenic FH [22]. Despite this, a consensus score has not yet been agreed upon. The different scores used differ from each other with regard to the number and compositions of the SNPs [23]. Also, some of the included variants show significantly different frequencies between populations, suggesting that a certain score might not be useful in all populations. Large-scale scores, which include hundreds to thousands of variants, retain much of the genome information but are difficult to handle in most clinical laboratories, as they require the use of high-throughput platforms and computer-intensive analysis. Small-scale scores, which include less than 100 SNPs, are easier to manage, and some have been designed to minimize the loss of information compared to that with large-scale scores. However, consensus in using such scores for clinical applications is lacking due to the heterogeneities between scores and different definitions of what constitutes a “high” score (top quartile, top decile, or another cutoff point). Thus, the main questions are (1) what set of SNPs is considered to drive the overall polygenic association in the population of study, and (2) how should a “high” score be defined?

In this study, we selected a sample of clinically diagnosed FH patients in Catalonia who participated in a previous genetic study about the classical candidate genes (*LDLR*, *APOB*, and *PCSK9*), and we calculated different weighted LDL-c-raising gene scores [16,17]. Our aims were to study the distribution of the wGSs in the clinically diagnosed FH patients to address the following in a clinical setting: (1) devise a small-scale genetic risk score that could be used to identify polygenic FH; (2) if not all the variants are informative, refine the number of SNPs in the score; (3) calculate a putative cutoff point for diagnosing polygenic hypercholesterolemia for our population.

## 2. Experimental Section

### 2.1. Subjects

We collected citrate blood or saliva samples from unrelated patients with biochemical and/or clinical traits of FH referred to our laboratory (Biochemistry Service, Hospital de la Santa Creu i Sant Pau, Barcelona, Spain). The characteristics of this population have been described previously [24]. In brief, the samples were sourced from 23 lipid clinic units around Catalonia participating in the Catalan Network of Lipids and Atherosclerosis (XULA in Catalan), a non-profit organization created to improve and assist clinical research in preventive cardiovascular medicine. The clinical diagnosis of FH was assessed after the application of the DLCN criteria [1,5], and clinical and/or biochemical data were provided in a summarized DLCN score form by the medical centers of origin. Although the genetic analysis of FH probands has been recommended only when DLCN ≥ 6, all the samples that arrived at our clinical laboratory were processed, since the suspicion of FH in some cases was not based only on the DLCN score. All the blood samples were obtained in accordance with the Helsinki Declaration of 1964, as revised in 2000. The Ethics Committee of the Hospital de la Santa Creu i Sant Pau reviewed and approved the study protocol (Project code IIBSP-DIS-2014-91, approved on 10/02/15), and only individuals who provided written informed consent were included. In the present study, we analyzed the first 670 patients of this collection of probands, including 250 mutation-positive and 420 mutation-negative FH patients. Also, 503 European samples from the 1000 Genomes (1000G) Project (www.internationalgenome.org, accessed on 18 October 2018), which include the genotype of 107 Spanish subjects, were used as controls. Since 107 was a low number to establish the percentiles with precision, the frequency in the Spanish population of each of the SNPs used in the score calculations was compared with the rest of the European populations. There were only differences with the Finnish population in two SNPs. Since only two of 44 (11 SNPs × 4) comparisons (4.5%) were significant, it was estimated that the heterogeneity of the European population for the analyzed variants allowed the use of the 503 European samples as controls in our study.

### 2.2. SNP Genotyping

Genomic DNA was extracted from leukocytes of peripheral whole blood samples using a QIAamp DNA Blood Mini Kit (Qiagen, Hilden, Germany), or from saliva collected in an Oragene^®^ DNA sample collection kit (DNA Genotek, Ottawa, ON, Canada) according to the manufacturer’s instructions. The genotypes of 11 of the 12 SNPs influencing LDL-c, as proposed by Talmud et al. (2013) [16], were genotyped using TaqMan^®^ SNP Genotyping Assays (Thermo Fisher Scientific, Waltham, MA, USA) on a CFX96™ Touch Real-Time PCR Detection System (Bio-Rad Laboratories, Inc., Hercules, CA, USA). The 12th SNP (rs11220462, *ST3GAL4* c.-61+18215G>A) was not analyzed, because a commercial TaqMan® assay was not available. The genotyped SNPs were *PCSK9* (rs2479409), *CELSR2-SORT1* (rs629301), *APOB* (rs1367117), *ABCG8* (rs4299376), *SLC22A1* (rs1564348), *HFE* (rs1800562), *MYLIP* (rs3757354), *NYNRIN* (rs8017377), *LDLR* (rs6511720), and *APOE* (rs429358 and rs7412). We calculated weighted LDL-c gene scores as the sum of the product of the number of copies of the effect allele by its corresponding effect size using the per-allele beta coefficients reported by the Global Lipid Genetics Consortium (GLGC) [25], as previously described [16,26] (Supplementary Table S1).

### 2.3. Statistical Analyses

The data are presented as means (standard deviation, SD) for continuous variables and frequencies for categorical variables. The plasma LDL-c of patients without lipid-lowering treatment was used in the categorized form according to the DLCN criteria [1] as follows: (1) <155 mg/dL, (2) 155–189 md/dL, (3) 190–249 mg/dL, (4) 250–329 mg/dL, and (5) >330 mg/dL. Categorical variables were compared using the chi-square test (or Fisher’s exact test when necessary). Differences in the distribution of continuous data between two groups were assessed with Student’s *t*-test except when the distribution departed from normality, in which case the Wilcoxon Mann–Whitney test was used. When more than two groups were involved, comparisons were assessed with the analysis of variance or the Kruskal–Wallis tests, depending on the normality and posterior post-hoc analyses. The optimal cutoff points were calculated using Youden’s J statistic [27]. Statistical calculations and graphic presentations were performed using R (v 3.6.3) [28] and RStudio (v 1.2.5033) [29]. *p* < 0.05 was considered to be statistically significant.

## 3. Result

### 3.1. Characteristics of the Population

The characteristics of the studied population have been previously reported [24] and are summarized in Table 1. In brief, of the 250 mutation-positive patients, 231 subjects presented a pathogenic variant in the *LDLR* gene, and 19 presented the *APOB* c.10580G>A variant. The patients with a positive genetic diagnosis (FH/M+) were younger than those without monogenic defects (*p* = 1.6 × 10^−4^) and presented a higher DLCN score (9.1) than the negative patients (7.1; *p* = 3.6 × 10^−14^) (Table 1). The percentage of FH/M+ were similar to the reported in other European and non-European populations [24]. The distribution of LDL-c ranks (*p* = 6.9 × 10^−14^) and family histories of children aged <18 years with LDL-c > 150 mg/dL (*p* = 3.1 × 10^−5^) were the components of the DLCN score that mainly contributed to the difference; both were higher in the FH/M+ patients. The frequency of first-degree relatives with LDL-c > 210 mg/dL was marginally higher in the FH/M+ patients (*p* = 0.072).

### 3.2. Genotyping

**Figure 1:**
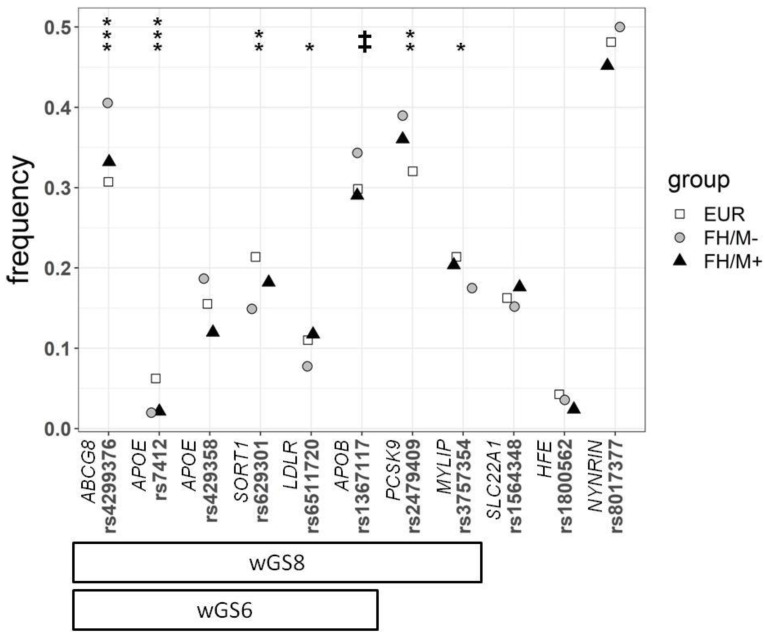
Frequency of the different SNPs used in this study for the 1000G European population (EUR), FH mutation-negative patients (FH/M−), and FH mutation-positive patients (FH/M+). The statistical significance differences between EUR and FH/M−are presented at the top. The *p* values were calculated by the chi-square test: ‡ *p* = 0.055; * *p* < 0.05; ** *p* < 0.01; *** *p* < 0.001. The two refinements of the weighted LDL-c gene score, calculated with 8 SNPs (wGS8), and the refinement of 6 SNPs proposed by Futema et al. [17] (wGS6), are shown at the bottom.

All the SNPs were in Hardy–Weinberg equilibrium, except *APOE* rs7412 in the FH/M− group (*p* = 0.0095). Not all the SNPs presented differences in frequency between the FH patients and controls (Figure 1). As the wGSs are based on the classical additive model, associations between each SNP and the different conditions were analyzed considering only this model. A stepwise multivariate logistic regression (Table 2) showed that seven SNPs contributed significantly and independently to the differences between the control European population and the FH/M− subjects. *ABCG8* (rs4299376), *APOE* (rs7412), *CELSR2* (*SORT1*) (rs629301), *PCSK9* (rs2479409), *LDLR* (rs6511720), *APOB* (1367117), and *MYLIP* (rs3757354) were independently associated with FH in decreasing level of significance. Thus, the wGS was calculated for 11 SNPs (wGS11), the six SNPs (wGS6) proposed by Futema et al. [17], and the set of seven SNPs significantly associated with FH in this study as well as *APOE* rs429358 to include the E2/E3/E4 genotypes (wGS8) (Figure 1).

### 3.3. Weighted Gene Scores

The three different wGSs were significantly higher in the FH/M− subjects with respect to the control European population and the FH/M+ patients (Table 3 and Supplementary Figure S1). In contrast, in the FH/M+ subjects only, wGS11 was significantly higher compared with the control European population (*p* = 0.033) (Table 3). FH/M+ subjects carrying a pathogenic variant in the *LDLR* gene appeared to present higher scores than the *APOB* mutation carriers (Supplementary Figure S2), but the difference was not statistically significant (Table 3).

#### 3.3.1. Estimation of the Number of Polygenic FH

**Figure 2:**
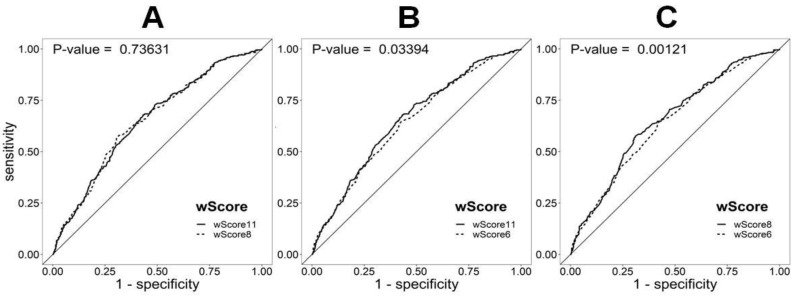
ROC curve analysis of the discrimination between FH mutation-negative patients (FH/M−) and the 1000G European population using different weighted LDL-c scores: (**A**) wScore11 (wGS11) vs. wScore8 (wGS8), (**B**) wScore11 (wGS11) vs. wScore6 (wGS6), and (**C**) wScore8 (wGS8) vs. wScore6 (wGS6). The difference between the AUC values of wGS11 (0.652) and wGS8 (0.654) was the only statistically non-significant difference. The AUC for the wScore6 (wGS6) was the lowest (0.640).

The suppression of non-significant SNPs (*SLC22A1* rs1564348, *HFE* rs1800562, and *NYNRIN* rs8017377) from the original set of 11 SNPs (wGS11) did not decrease the ability of wGS8 to discriminate between the FH/M− and control European populations (*p* = 0.74). This result is unlike that obtained by reducing the number of SNPs to the six SNPs (wGS6) proposed by Futema et al. [17] (*p* = 0.03) (Figure 2). Considering the values of the area under the ROC curve, wGS11 and wGS8 performed similarly, either in the whole FH/M− sample or considering only probable (DLCN = 6−8), probable+definite (DLCN ≥ 6), or definite (DLCN > 8) clinical diagnostic classes (Supplementary Table S2).

In the FH/M− group, 42.7% (172/403), 46.4% (187/403), and 47.1% (190/403) of the patients presented a score above the top quartiles of wGS11, wGS8, and wGS6 (Supplementary Table S3). The resulting thresholds from the ROC curves raised the proportions to 67.5% (272/403), 56.8% (229/403), and 64.5% (260/403) for wGS11 (threshold = 0.942), wGS8 (threshold = 0.834), and wGS6 (threshold = 0.705), respectively. Considering the different cutoffs, namely the 75th, 90th, and 95th percentiles, the fewer the SNPs included in the score, the higher the percentage of the FH/M− subjects with a score above the percentile (Supplementary Table S3). For the cutoff points calculated from the ROC curves, the wGS11 and wGS6 thresholds presented similar sensitivities, while wGS8 presented the lowest value. Thus, the most conservative score seems to be that of wGS8. Moreover, when considering only subjects with a clinical diagnosis of definite FH (DLCN > 8) and the 75th percentile as the cutoff point, wGS8 showed the highest sensitivity (51.1%).

#### 3.3.2. Association of wGS with Phenotype

With respect to the phenotype, none of the wGSs correlated with the DLCN score or the categorized cholesterol levels in any of the FH groups studied (FH/M−, FH/M+, and total FH). It should be noted that, despite that FH/M– patients presented on average higher scores than the FH/M+, the difference is mainly located in the range of LDL-c between 190–329 mg/dL, while in the extremes (< = 189 and > 330 mg/dL) the difference was smaller. Application of the Cochran–Armitage test showed no tendency to increase or decrease the proportion of patients with a score within the 75th percentile with increasing the categorized LDL-c level. A slight, but not significant, increase in wGS11 was observed in the FH/M− subjects with respect to their FH/M+ counterparts when the patients had a definite clinical FH diagnosis (Supplementary Figure S3). Multivariable logistic regressions of the different DLCN components and scores, adjusted for age and sex, showed that only a family history of a first-degree relative with premature coronary and/or vascular disease (component 1a in Table 1 or DLCN-1a) was negatively associated with the wGSs in all the groups. Specifically, wGS11 was negatively associated with DLCN-1a in the total FH (*p* = 0.015), FH/M− (*p* = 0.046), and FH/M+ (*p* = 0.069) groups. In the case of the FH/M+ patients, considering only the carriers of an *LDLR* mutation, the association was statistically significant (*p* = 0.015).

## 4. Discussion

Previously, we achieved a monogenic mutation confirmation in 36.8% of clinically diagnosed FH patients. This percentage of positives is slightly lower than that of previous studies focusing on Spain but similar to that of other European populations [24]. Also, the contribution of family history to the clinical diagnosis was lower compared with those reported in other studies in Spain. This could be due to differences in population recruitment, since our study sample included fewer patients from registries with extensive follow-ups [24]. In an attempt to determine whether our FH/M− patients have a polygenic form of FH as a result of the accumulation of LDL-raising alleles, we analyzed the distribution of an LDL-c-raising SNP score (wGS11) and compared it with two refinements of this score, one previously described (wGS6) and a new one including only the significantly associated SNPs (wGS8).

As expected, since the variants used in the scores are associated with an increase in the LDL-c plasma level, some of the SNPs presented differences in their frequencies between clinically diagnosed FH patients and the control population. The main difference in frequency was observed for *ABCG8* rs4299376 (Table 2 and Figure 1), which is consistent with previous findings indicating that a common single polymorphism in *ABCG5/8* modulates plasma lipid concentrations in FH patients and influences atherosclerosis risk [30,31]. The second variant showing significantly different frequencies between FH and the control groups was *APOE* rs7412. This variant was the only one absent from the Hardy–Weinberg equilibrium in the FH/M− group. This variant, p.Arg176Cys, constitutes the genetic basis of the Apoε2 allele and has been associated with higher apolipoprotein (Apo) E and lower plasma cholesterol concentrations in multiple studies [32,33]. Homozygous carriers for this variant are associated with dysbetalipoproteinemia or type III hyperlipoproteinemia (OMIM #617347), although only 1–4% of them develop the disease. In few studies, *APOE* E2/E2 genotype in mutation-negative FH patients was considered an exclusion criterion for polygenic FH diagnosis [34], but this is not a widely accepted rule. In our case, the lack of Hardy–Weinberg equilibrium is caused by the presence in the FH/M− group of two homozygous subjects: one with a DLCN of 9, including a first-degree relative with high LDL-c, a family history of coronary artery disease (CAD), and a personal history of LDL-c > 330 mg/dL, and the other with a DLCN of 6,a personal history of LDL-c = 250–329 mg/dL, and a family history of CAD. Both presented wGSs < 0 due to the negative effect of the *APOE* E2/E2 genotype.

It should be noted that, for the rest of the variants that presented significant differences between FH and the controls, namely *SORT1* rs629301, *PCSK9* rs2479409, *LDLR* rs6511720, *APOB* rs1367117, and *MYLIP* rs3757354, the differences were noted exclusively between the controls and FH/M− patients.

The calculated mean wGSs were significant higher in the FH/M− subjects than in the general European population and even the FH/M+ patients. This result is in agreement with those of previous studies, suggesting that plasma LDL-c is influenced by the accumulation of small-effect variants in genes influencing LDL metabolism. However, the distribution of wGSs was not uniform in the FH/M+ group, since carriers of an *LDLR* pathogenic mutation tend to present higher, although not statistically significant, genetic scores than *APOB* c.10580G >A carriers (Supplementary Figure S2). A previous study pointed out that higher scores in carriers of an *LDLR* mutation vs. carriers of an *APOB* mutation might indicate that some *LDLR* variants need other genetic defects to be pathogenic, while *APOB* mutations are sufficient to cause a defective LDL clearance [17].

As mentioned above, the consensus about using small-scale scores for clinical applications is currently lacking due to the heterogeneities among scores and the differing definitions of what would be considered as “high” (the 75th, 90th, or 95th percentiles) [23]. Further, the frequencies of the variants and their effects may vary in different populations, necessitating score testing in each population.

In our sample, the best discrimination between the FH/M− subjects and the control population was achieved with a score of 8 SNPs from seven different genes (AUC = 0.654, 95 confidence interval (CI): 0.62–0.69), which includes the most informative SNPs from the multivariate logistic regression. The cutoff point calculated in the ROC curves (0.834) was very similar to the wGS8 average in the FH/M− group (0.836) and slightly below the 75th percentile in the European population (0.864). These numbers corresponded to the highest specificity (69%) and the lowest sensitivity (57%) compared with the cutoff points obtained for wGS11 and wGS6. This finding, together with the fact that our refinement of wGS11 using 8SNPs (wGS8) presented a higher AUC, drove us to consider wGS8 as the best score and the 75th percentile as the best cutoff point in our population. This consideration was reinforced by the fact that wGS8 presented the higher sensitivity (51.1%) using the 75th percentile as the cutoff point and when a clinical FH diagnosis was definite (DLCN > 8). Thus, 66.4% (37.3 monogenic + 62.7 × 0.464 polygenic) of the total FH patients and 75.9% (50.7 monogenic + 49.3 × 0.511 polygenic) of the FH patients with a definite clinical diagnosis (DLCN >8) had an identified genetic cause.

Compared with other studies that used small-scale risk scores, our work presented one of the highest percentages of patients within the top quartile of the score distribution. In a sample of 1158 clinically diagnosed FH patients from seven countries, wGS6 of 36% of the FH/M−subjects was within the 75th percentile [17], and in a sample of 254 clinically diagnosed FH patients from Portugal, the percentage of FH/M− subjects with a wGS6 score within the 75th percentile equaled 38% [19], compared to 47.1% in our population. The corresponding percentage was 87.8% in a population from Israel, but the population size was only 67 patients [20]. With respect to studies that used the 90th percentile as the cutoff point, our research corresponds with the 19–20% of FH/M− patients within the top decile of the score distribution [16,21], but this proportion is less than that obtained (29%) for 313 FH patients from Canada [18]. The differences between the studies may be due to the disparities in the score distributions of the percentiles between populations. However, the estimated percentiles for wGS11 in the 1000G European samples (Supplementary Table S4) do not seem to be significantly different from those of the Whitehall II cohort from the UK [16], even considering that we did not include one of the SNPs.

As previously described [24], our population sample was referred to our laboratory from 23 lipid clinic units around Catalonia, and the clinical and biochemical data were provided in a summarized DLCN score. Thus, we did not have access to raw data such as exact plasma LDL-c concentrations, and we could only study the associations with the different categorical components of the DLCN score. In our sample, a personal history of CAD was four-fold lower than the family history, with no differences between the FH/M− and FH/M+ subjects (Table 1). Also, wGSs were not associated with the DLCN clinical score, the categorized LDL-c plasma concentration, or personal history of CAD. An association between different wGSs and lipid traits was previously described [13,14,25,35,36,37], although wGSs for LDL-c did not present an increase in their plasma concentrations with age [35,36,38], while another study reported a lack of association with LDL-c [39]. Since we did not have the exact values of the LDL-c plasma concentrations, the lack of association with the score should be considered with caution. An independent study in FH families from Spain showed that the heritability of LDL-c in FH/M− subjects (0.32) was lower than that in their FH/M+ counterparts (0.61) [40]. This may explain the tendency for the lower frequency of having a first-degree relative with LDL-c > 210 mg/dL in FH/M− subjects than in FH/M+ patients (*p* = 0.073) and the negative association of wGS11 with a family history of CAD.

It is unknown whether wGSs can be translated to individual patients and, therefore, whether they are of any use in clinical practice [39]. At present, the DLCN criteria have been modified to include genetic analysis. The presence of a pathogenic variant in the *LDLR*, *APOB*, or *PCSK9* genes is sufficient to diagnose FH as virtually definite [41,42] without requiring further factors. In contrast, the large overlap of the wGSs distributions between the FH and control subjects adds to the difficulty of considering the polygenic score as an independent diagnostic criterion. Therefore, the accumulation of variants with small effects seems to require the presence of other factors to develop the FH phenotype. It should be noted that genetic scores include variants associated with plasma LDL-c in GWASs studies, but an association between an SNP and a trait does not imply a direct cause–effect relationship [43]. GWASs initially include only common variants, with minor allele frequency (MAF) >5%. However, today, low-frequency variants (0.1% < MAF ≤ 5%) have been included in some GWASs. These low-frequency variants increase the proportion of LDL-c variance explained by common variants from 16.3% to 19.5% [44], while variants with known functional impacts on lipids explain 6.7% of the variance. Also, a recent deep-coverage whole-genome sequencing study detected 697 variants associated with LDL-c, distributed at 10 loci, with a genome-wide significance ≥ 5.0 × 10^−8^ [45]. However, small-scale genetic scores usually include only the highest significant variant from each locus, which sometimes differs between GWASs, probably due to differences in frequencies between populations. As we pointed out above, analyzing a high number of SNPs, as in large-scale scores, increases the cost and the complexity of the analysis, since it involves the use of a high throughput platform and intense bioinformatic analysis, which are unavailable in most clinical laboratories. However, a decrease in the number of SNPs implies a reduction of information [17]. Thus, a balance between utility and cost-effectiveness needs to be achieved.

The initial 12-SNP score proposed by Talmud et al. (2013) [16] was based on the LDL-c raising alleles identified by the GLGC [25] in a meta-analysis of 46 lipid GWASs comprising over 100,000 individuals of European descent from different countries. An addition of 21 SNPs to the 12-SNP score failed to improve results in a cohort of European ancestry from the UK [17]. However, recently, a study in a cohort of white Americans using a small-scale score including 36 SNPs, also from the GLGC, was able to explain 30% more variability in the LDL-c trait than the 12-SNP score [46]. This 36-SNP and the expanded 33-SNP scores used different selection methods and, as a result, only shared nine variants from seven genes, and had in common three more genes but including different variants. Thus, current small-scale wGSs are not informative enough to be used worldwide and should be adapted to each population. More studies are needed to design more specific wGSs that allow diagnosis of polygenic hypercholesterolemia. In this sense, one of the limitations of this study is that the population used as a control was not entirely of the same origin as that of the patients, although the SNPs analyzed did not show significant differences between the five populations that made up the control population.

It cannot be ruled out that the scores used in our study did not identify all patients with polygenic FH. A considerable percentage of the remaining 53.6% of FH/M– patients could also be polygenic FH with combinations of other variants in the same or in different genes. Complex forms of FH due to the interaction of a set of variants with environmental factors cannot be ruled out either. These forms will likely be more difficult to identify since, in the absence of the triggering environmental factor, the genotype would not be associated with an altered lipid profile, thereby reducing the genetic difference with a healthy control population.

Lastly, the possibility that 5–20% of suspected FH are cases of genetically-driven high levels of lipoprotein(a) (Lp (a)) has been pointed out [47], since the Friedewald formula does not discriminate between cholesterol linked to LDL or Lp (a). A study of a cohort of 933 clinically diagnosed FH patients from Spain [48] showed that 28.9% of FH/M- subjects had also hyperLp (a), defined as Lp (a) plasma level ≥95th percentile of the reference population. However, when LDL-c plasma level was corrected for the cholesterol linked to Lp (a), the proportion of FH/M- patients with isolated hyperLp (a) was 6.4%. The possibility of pathological variants in atypical genes or confusion with other pathologies, like sitosterolemia, is estimated to represent less than 2% [47].

## 5. Conclusions

Weighted LDL-c-raising SNP scores can still be improved and must be adapted to each population. Our data indicate that a score based on eight SNPs and the 75th percentile cutoff point may identify patients with polygenic FH in Catalonia and, together with the 37.3% of patients previously identified with monogenic FH, wGS8 would allow for the assignment of a genetic cause to 66.4% of the clinically diagnosed FH patients.

## Figures and Tables

**Table 1:** Characteristics of the population studied according to the absence (FH/M–) or presence (FH/M+) of a mutation.

	Total	FH/M−	FH/M+	(*p* Value) ^1^
N (%)	670	420 (62.7%)	250 (37.3%)	
Sex				
Males (%)	46.0%	46.4%	45.2%	ns (0.82)
Females (%)	54.0%	53.6%	54.8%	
Age (yrs) ^2^	44.8 (13.0)	46.4 (12.4)	42.3 (13.7)	*** (1.6 × 10^−4^)
DLCN ^2^	7.84 (3.4)	7.09 (2.6)	9.09 (4.1)	*** (3.6 × 10^−14^)
DLCN <3	0.9%	0.7%	1.2%	*** (3.2 × 10^−7^)
DLCN 3–5 (possibly)	17.4%	21.3%	10.9%	
DLCN 6–8 (probably)	50.0%	53.6%	44.0%	
DLCN >8 (definite)	31.8%	24.4%	44.0%	
Components of DLCN				
Family history				
1a. first degree relative with premature ^3^	42.0%	43.5%	39.6%	ns (0.44)
coronary and/or vascular disease				
1b. first degree relative with	63.5%	60.3%	68.5%	ns (0.073)
LDL-c > 210mg/dL				
1a and/or 1b	81.7%	80.2%	83.1%	ns (0.56)
2a. first degree relative with tendinous	6.7%	5.1%	9.4%	ns (0.072)
xanthomata and/or arcus cornealis				
2b. children aged <18 yrs with	27.0%	20.8%	37.3%	*** (3.1 × 10^−5^)
LDL-c >150 mg/dL				
2a and/or 2b	34.6%	27.7%	45.8%	*** (1.1 × 10^−5^)
Clinical history				
3a. patients with premature ^3^	9.3%	9.6%	8.9%	ns (0.89)
coronary artery disease				
3b. patients with premature ^3^	4.7%	4.9%	4.4%	ns (0.94)
cerebral or peripheral vascular disease				
Physical examination				
4a. tendinous xanthomata	15.4%	13.7%	18.2%	ns (0.17)
4b. arcus cornealis before 45 years of age	19.0%	18.4%	20.0%	ns (0.70)
LDL-cholesterol				
5a. LDL-c > 330 mg/dL	10.3%	3.8%	21.2%	*** (6.9 × 10^−14^)
5b. LDL-c 250–329 mg/dL	39.7%	37.1%	44.0%	
5c. LDL-c 190–249 mg/dL	33.4%	40.5%	21.6%	
5d. LDL-c 155–189 mg/dL	3.1%	3.8%	2.0%	

^1^ Chi-square testing for frequency comparison, or independent-samples *t*-test for age and DLCN score. *ns* = not significant; * *p* < 0.05; ** *p* < 0.01; *** *p* < 0.001. ^2^ mean (standard deviation). ^3^ premature: men aged <55 years, women aged <60 years.

**Table 2:** SNPs associated to FH in the stepwise logistic regression.

Gene	Id	OR	95% C.I.	*p* Value	
*ABCG8*	rs4299376	1.51	(1.23–1.86)	7.7 × 10^−5^	***
*APOE*	rs7412	0.33	(0.18–0.56)	9.9 × 10^−5^	***
*CELSR2 (SORT1)*	rs629301	0.62	(0.48–0.80)	0.0002	***
*PCSK9*	rs2479409	1.42	(1.16–1.75)	0.0009	***
*LDLR*	rs6511720	0.70	(0.50–0.97)	0.0369	*
*APOB*	rs1367117	1.24	(1.01–1.52)	0.0409	*
*MYLIP*	rs3757354	0.78	(0.61–0.99)	0.0413	*

* *p* < 0.05; *** *p* < 0.001.

**Table 3:** Differences in calculated weighted LDL-c gene score between FH patients without an identified mutation (FH/M−), and monogenic FH (FH/M+) patients with and identified mutation in the *LDLR* gene (FH/M+ *LDLR*), FH/M+ patients with an identified mutation in the *APOB* gene (FH/M+ *APOB*), and the 1000G European population (EUR) groups.

	FH/M−	FH/M+	FH/M+–*LDLR*	FH/M+–*APOB*	EUR
wGS11					
FH/M−	**0.990 (0.18)**				
FH/M+	*** (*p* = 3.6 × 10^−6^)	**0.928 (0.18)**			
FH/M+–*LDLR*	*** (*p* = 4.2 × 10^−5^)	-	**0.931 (0.17)**		
FH/M+–*APOB*	* (*p* = 0.050)	-	ns (*p* = 0.645)	**0.885 (0.18)**	
EUR	*** (*p* = 8.7 × 10^−15^)	* (*p* = 0.033)	ns (*p* = 0.066)	ns (*p* = 0.796)	**0.887 (0.21)**
wGS8					
FH/M−	**0.836 (0.18)**				
FH/M+	*** (*p* = 4.7 × 10^−7^)	**0.769 (0.17)**			
FH/M+–*LDLR*	*** (*p* = 1.0 × 10^−5^)	-	**0.773 (0.17)**		
FH/M+–*APOB*	* (*p* = 0.015)	-	ns (*p* = 0.336)	**0.721 (0.17)**	
EUR	*** (*p* = 2.7 × 10^−15^)	ns (*p* = 0.072)	ns (*p* = 0.126)	ns (*p* = 0.604)	**0.732 (0.21)**
wGS6					
FH/M−	**0.734 (0.18)**				
FH/M+	*** (*p* = 1.9 × 10^−6^)	**0.673 (0.17)**			
FH/M+–*LDLR*	*** (*p* = 3.1 × 10^−5^)	-	**0.676 (0.17)**		
FH/M+–*APOB*	* (*p* = 0.040)	-	ns (*p* = 0.614)	**0.635 (0.16)**	
EUR	*** (*p* = 8.9 × 10^−13^)	ns (*p* = 0.148)	ns (*p* = 0.291)	ns (*p* = 0.658)	**0.641 (0.21)**

In bold, weighted LDL-c score expressed as mean (SD). * *p* < 0.05; ** *p* < 0.01; *** *p* < 0.001; ns, not significant.

## Supplementary Materials

The following are available online at https://www.mdpi.com/2227-9059/8/9/353/s1, Figure S1: Distribution of the different wGS in each group, Figure S2: Boxplot of the calculated different weighted LDL-c gene scores in the different groups, Figure S3: Distribution of the wGS11 depending on the DLCN score and mutation status, Table S1: SNPs associated with LDL-c plasma level used in the study, Table S2: Discrimination values between FH/M- and control populations, Table S3: Percentiles of the different wGSs in the European population.
