# Supplementary material for: Polygenic Markers in Patients Diagnosed of Autosomal Dominant Hypercholesterolemia in Catalonia: Distribution of Weighted LDL-c-Raising SNP Scores and Refinement of Variant Selection
Source: Biomedicines. 2020 Sep 15;8(9):353. doi: 10.3390/biomedicines8090353 (PMC7554998; doi:10.3390/biomedicines8090353)
Supplement: Supplementary file 1 [file biomedicines-08-00353-s001.pdf]

Supplemental data

Table S1: SNPs associated with the LDL-c plasma level used in the study.

gene	ID	position[a]	cDNA	protein	change[b]	Ancestral	MA[c]	effect size[d]
PCSK9	rs2479409	1:55038977	c.–861G>A	(upstream)	G>A	A	G	0.052
CELSR2 (SORT1)	rs629301	1:109275684	c.*1635G>T	(3'UTR)	G>T	T	G	−0.146
APOB	rs1367117	2:21041028	c.293C>T	p.Thr98Ile	G>A	G	A	0.105
ABCG8	rs4299376	2:43845437	c.166–718G>T	(intronic)	G>T	T	G	0.071
SLC22A1	rs1564348	6:160157828	c.1599–688T>C	(intronic)	T>C	T	C	0.051
HFE	rs1800562	6:26092913	c.845G>A	p.Cys282Tyr	G>A	G	A	−0.057
MYLIP	rs3757354	6:16127176	c. −2147C>T	(upstream)	C>T	C	T	−0.037
NYNRIN	rs8017377	14:24414681	c.2932G>A	p.Ala978Thr	G>A	G	A	0.03
LDLR	rs6511720	19:11091630	c.67+2015G>T	(intronic)	G>T	G	T	−0.181
APOE	rs429358	19:44908684	c.388T>C	p.Cys130Arg	T>C	C	C	
APOE	rs7412	19:44908820	c.526C>T	p.Arg176Cys	C>T	C	T	
APOE genotype								
	E2/E2	E2/E3	E2/E4	E3/E3	E3/E4	E4/E4		
effect size[d]	-0.9	−0.4	−0.2	0.0	0.1	0.2		

[a], positions are relative to genome assembly GRCh38.p2. [b], alleles are designated with respect to the forward strand. [c], minor allele (MA). [d], effect size due to a single copy of the minor allele for LDL-c in mmol/L, taken from Supplementary Table S1 in Futema et al. [17].

Table S2: Differences in the area under the receiver operating characteristic (AROC) curve between the control European population and familial hypercholesterolemia mutation negative patients (FH/M−).

Score	AROC (95%CI)	wGS11[1]	wGS8[2]
**All FH/M−**			
wGS11	0.652 (0.617–0.687)		
wGS8	0.654 (0.619–0.690)	ns (p = 0.736)	
wGS6	0.640 (0.604–0.675)	* (p = 0.034)	** (p = 0.0012)
**probable + definite (DLCN ≥ 6)**			
wGS11	0.649 (0.611–0.687)		
wGS8	0.652 (0.614–0.690)	ns (p = 0.703)	
wGS6	0.639 (0.600–0.677)	ns (p = 0.077)	** (p = 0.0056)
**probable (DLCN 6–8)**			
wGS11	0.637 (0.594–0.680)		
wGS8	0.639 (0.596–0.682)	ns (p = 0.943)	
wGS6	0.626 (0.583–0.669)	ns (p = 0.114)	* (p = 0.0257)
**definite (DLCN > 8)**			
wGS11	0.676 (0.618–0.735)		
wGS8	0.681 (0.623–0.738)	ns (p = 0.452)	
wGS6	0.667 (0.610–0.725)	ns (p = 0.235)	* (p = 0.0237)

[1], comparison with the AROC obtained with wGS11. [2], comparison with the AROC obtained with wGS8. *, $p < 0.05$; **, $p < 0.01$; ***, $p < 0.001$; ns, not significant

Table S3: Discrimination between the control European population (EUR) and familial hypercholesterolemia mutation negative patients (FH/M−), depending on different cutoff values.

						All FH/M−		probable (DLCN 6–8)		probable + definite (DLCN ≥ 6)		definite (DLCN > 8)
**Score**	**threshold[1]**	**value**	**n[2]**	**specificity % (95CI)**	**n[2]**	**sensitivity % (95CI)**	**n[2]**	**sensitivity % (95CI)**	**n[2]**	**sensitivity % (95CI)**	**n[2]**	**sensitivity % (95CI)**
Total individuals			503		403		218		312		94	
wGS11	ROC	0.942	217	56.9 (52.7–61.2)	272	67.5 (62.8–72.0)	147	67.4 (61.5–73.4)	211	67.6 (62.5–72.4)	64	68.1 (58.5–77.7)
	75th	1.027	128	74.6 (71.0–78.1)	172	42.7 (38.0–47.4)	88	40.4 (33.9–46.8)	132	42.1 (36.9–47.8)	44	46.8 (37.2–56.4)
	90th	1.130	51	89.9 (87.1–92.2)	77	19.1 (15.4–23.1)	39	17.9 (12.8–22.9)	64	20.5 (16.0–25.0)	25	26.6 (18.1–36.2)
	95th	1.178	27	94.6 (92.6–96.6)	54	13.4 (10.2–16.9)	27	12.4 (8.3–17.0)	45	14.4 (10.6–18.3)	18	19.1 (11.7–27.7)
wGS8	ROC	0.834	156	69.0 (65.0–72.8)	229	56.8 (52.1–61.8)	121	55.5 (48.6–61.9)	177	56.7 (51.3–62.2)	56	59.6 (48.9–69.1)
	75th	0.864	126	75.0 (71.2–78.7)	187	46.4 (41.7–51.4)	93	42.7 (35.8–49.1)	141	45.2 (39.4–50.6)	48	51.1 (40.4–60.6)
	90th	0.971	51	89.9 (87.3–92.4)	84	20.8 (16.9–25.1)	39	17.9 (12.8–22.9)	66	21.2 (16.7–26.0)	27	28.7 (20.2–38.3)
	95th	1.010	26	94.8 (92.8–96.6)	57	14.1 (10.9–17.6)	29	13.3 (9.2–17.9)	48	15.4 (11.2–19.6)	19	20.2 (12.8–28.7)
wGS6	ROC	0.705	216	57.1 (52.9–61.2)	260	64.5 (59.8–69.2)	136	62.4 (56.0–68.8)	198	63.5 (58.3–68.9)	62	66.0 (56.4–76.6)
	75th	0.759	147	70.8 (66.8–74.8)	190	47.1 (42.2–52.1)	99	45.4 (38.5–51.8)	146	46.8 (41.0–52.2)	47	50.0 (40.4–59.6)
	90th	0.864	54	89.3 (86.5–92.0)	83	20.6 (16.6–24.6)	37	17.0 (11.9–22.0)	64	20.5 (16.3–25.3)	27	28.7 (19.1–38.3)
	95th	0.930	35	93.0 (90.7–95.2)	61	15.1 (11.7–18.6)	31	14.2 (9.6–19.3)	50	16.0 (11.9–20.2)	19	20.2 (11.7–28.7)

[1], cutoff value obtained from the receiver operating characteristic (ROC) analysis, and the 75th, 90th and 95th percentiles of the EUR population. [2], number of individuals above the cutoff value or in the corresponding percentile.

Table S4: Weighted LDL-c-raising SNPs score percentiles in the 1000 genomes European population.

	wGS11	wGS8	wGS6
minimum	−0.06	−0.23	−0.36
maximum	1.44	1.26	1.14
mean (SD)	0.89 (0.21)	0.73 (0.21)	0.64 (0.21)
Percentiles (range)			
Decile 1	−0.06 to 0.62	−0.23 to 0.46	−0.36 to 0.36
Decile 2	0.62 to 0.73	0.46 to 0.58	0.36 to 0.51
Quartile 1	−0.06 to 0.78	−0.23 to 0.63	−0.36 to 0.54
Decile 3	0.73 to 0.82	0.58 to 0.66	0.51 to 0.58
Decile 4	0.82 to 0.87	0.66 to 0.72	0.58 to 0.63
Decile 5	0.87 to 0.92	0.72 to 0.76	0.63 to 0.65
Decile 6	0.92 to 0.96	0.76 to 0.80	0.65 to 0.73
Decile 7	0.96 to 1.00	0.80 to 0.85	0.73 to 0.75
Quartile 3	0.92 to 1.03	0.76 to 0.86	0.65 to 0.76
Decile 8	1.00 to 1.05	0.85 to 0.90	0.75 to 0.83
Decile 9	1.05 to 1.13	0.90 to 0.97	0.83 to 0.86
Decile 10	1.13 to 1.44	0.97 to 1.26	0.86 to 1.14

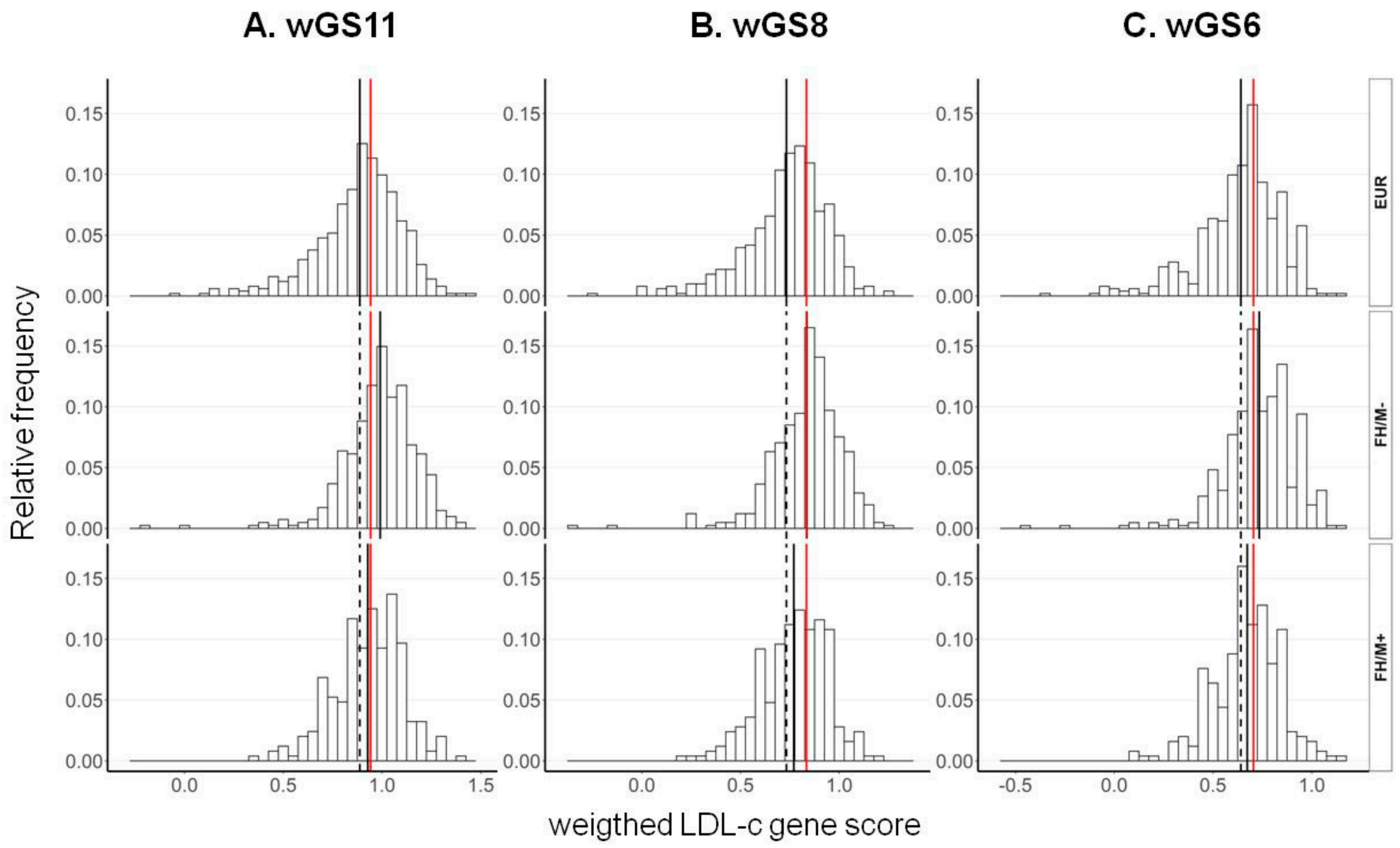

Figure S1: Distribution of weighted LDL-c gene scores for the 1000G European population (EUR), FH mutation-negative patients (FH/M−), and FH mutation-positive patients (FH/M+). Different distributions correspond to weighted LDL-c gene scores calculated with (**A**) 11 SNPs (wGS11), (**B**) 8 SNPs associated with FH in this study (wGS8), and (**C**) the refinement of 6 SNPs proposed by Futema et al. [17] (wGS6). The mean of each group is represented by a solid black line. The mean of the EUR group is denoted by a dashed black line in the FH groups. The calculated threshold is represented by a solid red line.

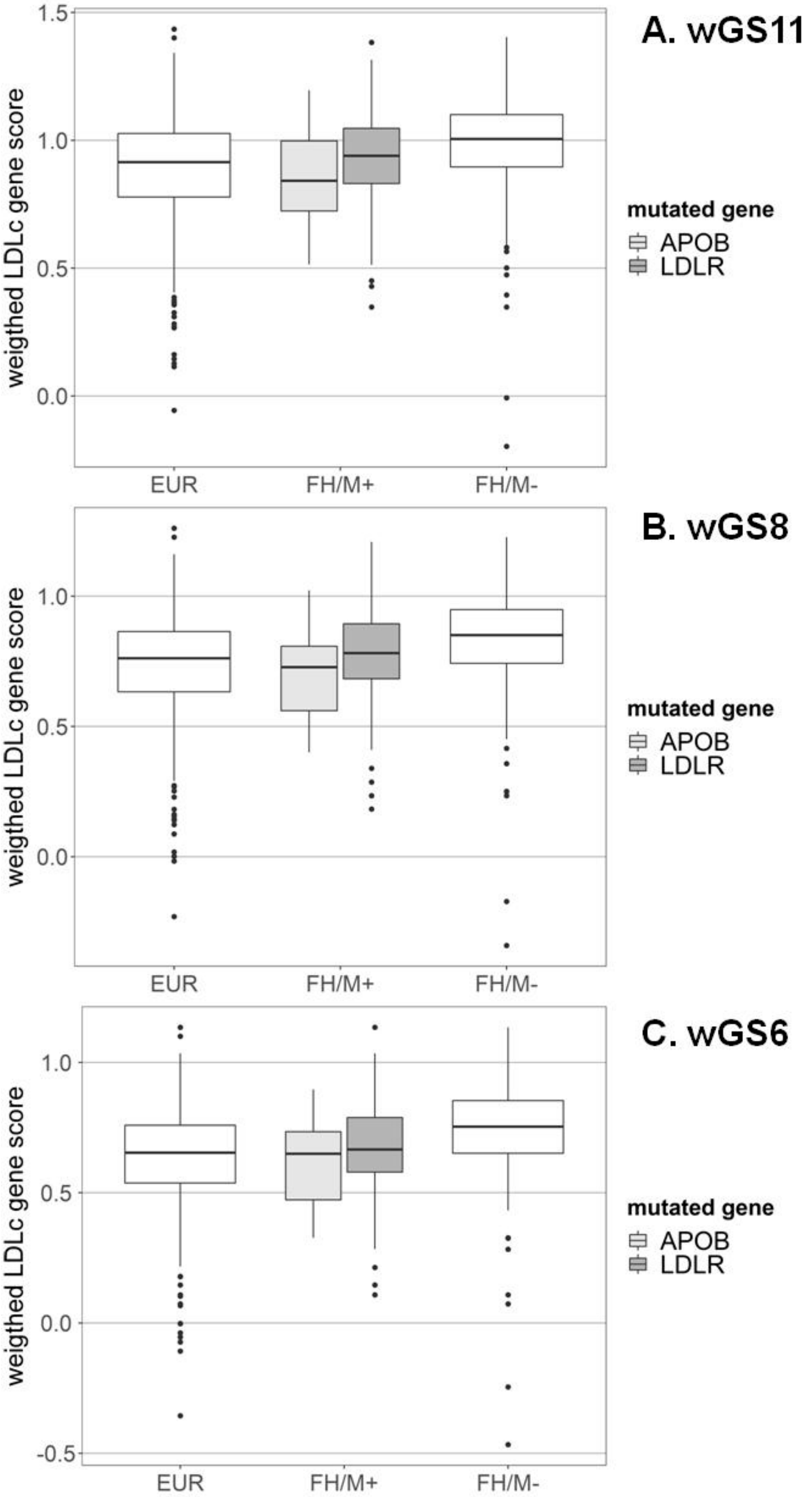

Figure S2: Boxplot of the calculated different weighted LDL-c gene scores in the different groups: EUR, 1000G European population; FH/M+, FH carriers of a mutation in the *APOB* or *LDLR* genes; FH/M−, FH patients without a mutation in the candidate genes. (**A**) weighted gene score for 11 SNPs, (**B**) weighted gene score for 8 SNPs, and (**C**) weighted gene score for 6 SNPs.

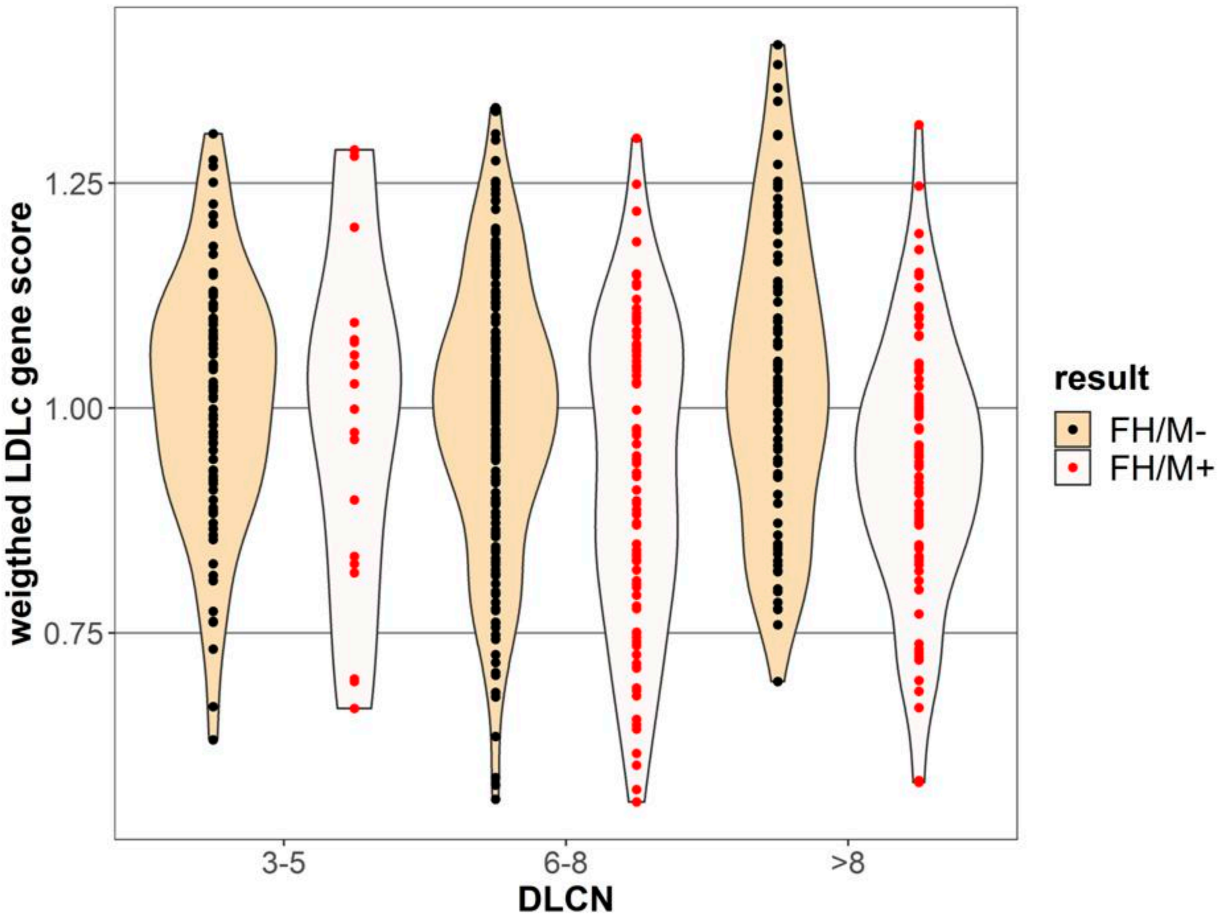

Figure S3: Distribution of the calculated weighted LDL-c gene score based on 11 SNPs (wGS11) depending on the clinical diagnostic and mutation status. Patients are divided into FH carriers of a mutation in the *APOB* or *LDLR* gene (FH/M+) and FH patients without a mutation in the candidate genes (FH/M−). Clinical diagnostics are divided in the DLCN score ranges of 3–5 (possibly FH), 6–8 (probably FH), and >8 (definite FH).
